# Importance of TP53 codon 72 and intron 3 duplication 16 bp polymorphisms and their haplotypes in susceptibility to sarcopenia in Iranian older adults

**DOI:** 10.1186/s12877-022-02765-6

**Published:** 2022-02-05

**Authors:** Nima Montazeri-Najafabady, Mohammad Hossein Dabbaghmanesh, Nasrin Nasimi, Zahra Sohrabi, Alireza Estedlal, Naeimehossadat Asmarian

**Affiliations:** 1 Endocrinology and Metabolism Research Center, Shiraz University of Medical Sciences, Shiraz, Iran; 2 Nutrition Research Center, Shiraz University of Medical Sciences, Shiraz, Iran; 3 Anesthesiology and Critical Care Research Center, Shiraz University of Medical Sciences, Shiraz, Iran

**Keywords:** Sarcopenia, p53, Polymorphism, Body composition, Lipid profile

## Abstract

**Background:**

Sarcopenia is described as age-related progressive skeletal muscle failure that results in marked reduction in the patient’s independence and life quality. In this study, we explored the association of TP53 exon 4 Arg72pro (rs1042522) and Intron 3 16-bp Del/Ins (rs17878362) polymorphisms and their haplotypes with sarcopenia, anthropometric, body composition and biochemical parameters.

**Methods:**

A total of 254 older individuals (65 sarcopenic and 189 healthy) were recruited in this research and genotyped by PCR–RFLP. Linear regression was applied to find the correlation between TP53 polymorphism, and biochemical and anthropometric parameters. The correlation between TP53 polymorphism and haplotypes and the risk of sarcopenia was investigated by logistic regression.

**Results:**

Arg/Pro genotype carriers was at a lower (OR_adj_ = 0.175, 95% CI = 0.068 – 0.447; *P* < 0.001) risk of sarcopenia compared to the Arg/Arg group. In haplotypes analysis, Arg-Ins (OR_adj_: 0.484, 95% CI = 0.231 – 1.011, *P* = 0.043) and Pro-Ins (OR_adj_: 0.473, 95% CI = 0.210 – 1.068, *P* = 0.022) haplotypes showed decreased risk of developing sarcopenia. Moreover, in the case of codon 72 polymorphism, skeletal muscle mass, appendicular lean mass (ALM), skeletal muscle mass index (SMI), hand grip strength and Triglycerides, for Intron 3 16-bp Del/Ins polymorphism, albumin, calcium, cholesterol, and LDL were different, and for the haplotypes, skeletal muscle mass, SMI, ALM, HDL and triglycerides were significantly different between groups.

**Conclusions:**

We suggested that the Arg/Pro genotype of the codon 72 polymorphism in exon 4 of TP53, and Arginine-Insertion and Proline-Insertion haplotypes might decrease the risk of sarcopenia in Iranian older adults.

## Introduction

The European Working Group on Sarcopenia in Older People (EWGSOP) defined sarcopenia as a progressive and a generalized skeletal muscle disorder that involves the accelerated loss of muscle mass and function [1]. Losses of muscle strength, muscle mass, and muscle quality (muscle failure) [1] and subsequent decline in performance of daily-life activities is particularly associated with a marked reduction in the patient’s independence and increased risk of falls and hip fractures [2, 3] which results in vicious cycle that predisposes patients to more functional disability [4], hospitalization [5], decreased life quality, and augmented risk of general morbidity and all-cause mortality [4]. In addition, recent literature revealed associations between sarcopenia and incidence of stroke [6], hypertension [7], depression [8] and higher prevalence of cognitive impairment [9].

Regarding the determined high heritability of skeletal muscle related traits [10, 11], there is a recent growing motivation towards the investigation of the genetic variation of muscle traits among individuals. Notwithstanding, the genetic underpinnings of skeletal muscle traits still remain largely unknown and the genetic aspects of sarcopenia are even less clear. Limited genetic linkage-analysis and Genome-wide association studies identified thyrotropin-releasing hormone receptor (TRHR) gene, the Angiotensin converting enzyme (ACE) gene, α-actinin-3 (ACTN3), ciliary neurotrophic factor (CNTF) gene, myostatin-related genes (MSTN), vitamin D receptor (VDR) gene, and androgen receptor (AR) gene to contribute to muscle aging, muscle strength, and mass with relevance to sarcopenia [11]. Moreover, with reference to the complex molecular mechanisms of skeletal muscle homeostasis, atrophy, and aging that give rise to sarcopenia [12, 13], several other hub genes of interest such as tumor suppressor protein 53 (TP53) gene, insulin growth factor 1 (IGF-1) gene, and interleukin-6 (IL-6) gene are proposed for better understanding of the pathogenesis of sarcopenia [14].

The TP53 gene codes for a 53-kDa protein involved in various aspects of skeletal muscle cell function, differentiation, and physiology. First and foremost, Tp53 is the main regulator of cell cycle, apoptosis, and genomic stability and therefore act as a threshold regulator of cellular homeostasis [15, 16]. Second, is the role of TP53 in cell senescence [17, 18]. The rate of protein synthesis in myocytes and regenerative function of muscle stem cells known as muscle satellite cells decline during aging; accordingly results in muscle atrophy and progressive age-related loss of muscle mass [19].

It is known in the literature that the alterations in transcriptional activity of TP53 and its wide array of protein–protein interactions can result in alterations in the indicated regulatory functions of this gene and subsequently strengthen the proposed putative role of Tp53 in sarcopenia [20–22]. TP53 polymorphism have been widely studied for the associations with different cancers and reports suggested of changes in transcriptional and thus biologic actions of various variants of this gene. Two of the most studied polymorphic variants are codon 72 Arg/Pro (CGC to CCC, rs1042522), IVS3 16 bp Del/Ins (rs17878362) [23].

Codon 72 is located within a proline-rich region that is necessary for the protein p53 to induce apoptosis. Evidence from in vitro studies suggests that these two protein forms, Arg72 and Pro72, have distinct activities and effects on regulating cell growth. Protein with Arg72 was reported to have up to 15-fold increased apoptotic ability compared to Pro72 variant [20].

Besides, the most common intronic variation in TP53 is a 16-base pair (bp) 11 insertion/deletion in intron 3 (rs17878362, consisting of one copy (A1 allele) or two copies (A2 allele) of the sequence ACCTGGAGGGCTGGGG, PIN3 (polymorphism in intron 3 (rs17878362)) [24]. Gemignani et al. found that this 16 bp insert allele was associated with lower levels of TP53 transcripts, suggesting that polymorphism causes an alteration in mRNA processing [25].

On the account of the proven importance and prevalence of sarcopenia and due to limited published data about the relation of TP53 polymorphisms and sarcopenia, we explored the genotype and allelic frequency of two most studied polymorphisms of codon 72 polymorphism in exon 4 (rs1042522) and Intron 3 16 bp Del/Ins (rs17878362) polymorphism and their haplotypes in sarcopenia among Iranian community-dwelling older adults. In our last study, we found the association between TP53 rs1625895 polymorphism and the risk of sarcopenic obesity in Iranian older adults [26]. In addition, we evaluated the association of anthropometric, body composition and biochemical parameters with genetic variants of TP53. Identifying the complex interactions between genetic variants and their association with sarcopenia can enable us to yield better screening programs, ascertain more precise prognosis, and inventing individualized treatment approaches [10, 20].

## Materials and Methods

### Study population

The present study is a cross-sectional investigation to explore the genetic variation of TP53 polymorphisms. The current study is a subgroup of the cross-sectional, population-based geriatric health examination survey (from August 2017 to February 2018) assessing the frequency of sarcopenia and its determinants among Iranian elderly individuals [27]. 254 older adults were selected using stratified, multistage sampling. 65 individuals were sarcopenia patients and 189 were controls. The inclusion criteria were independent physically active older adults by the age 65 and older with no past medical history for severe cardiac, pulmonary, musculoskeletal disease, no nervous system disorders such as Parkinson or history of stroke and no malignancies or any other organ failures. All procedures were performed in the study involving human participants were in accordance with The Code of Ethics of the World Medical Association (Declaration of Helsinki) for experiments involving humans. The research protocol was submitted, evaluated and confirmed at Shiraz University of Medical Sciences, ethics committee by ethic code: IR.sums.rec.98–01-01–21,727. This study has been performed in accordance with the Declaration of Helsinki. The written informed consent for the use of samples was obtained from all participants.

### Baseline characteristics

Age was determined by the self-report questionnaire obtained from the study population. Body weight, height, waist, arm, and calf circumferences were measured. Body Mass Index (BMI) was measured using the following equation: BMI (kg/m^2^) = weight (kg) / [height (m)] ^2^.

### Diagnostic measures

Sarcopenia was defined and stated among the participants according to the European Working Group on Sarcopenia in Older People (EWGSOP) algorithm [1]. This syndrome was characterized by progressive and generalized loss of skeletal muscle mass and muscle strength which is the most reliable measure of muscle function.

Skeletal muscle mass was assessed using a segmental multi-frequency Bioelectrical Impedance Analysis (BIA) In Body S10 analyzer (BioSpace Co., Ltd., South Korea) which measured segmental lean body mass, Fat-Free Mass (FFM), and body fat mass. Skeletal Muscle Mass Index (SMI) was defined as a sum of arms and legs muscle mass (called Appendicular Lean Mass (ALM)) divided by the height squared (m^2^) and values less than 7.0 kg/m2 for males and 5.7 kg/m2 for females were reflected as low muscle mass [28].

Muscle function was appraised using Hand grip Strength (HGS) and usual Gait Speed (GS). HGS was measured using a hydraulic hand dynamometer (model MSD, Sihan, Korea) in sitting position. The mean of three measurements for both hands was calculated and HGS less than 26 kg for males and 18 kg for females were considered as low muscle strength. GS was also assessed by a 4 m of independent walking and GS less than 0.8 m/s was considered as a low physical performance [28].

### Biochemical measurements

Blood samples were gathered after a 12 h fasting in hormone laboratory of the endocrinology and metabolism research center at Nemazee Hospital (an educational hospital affiliated to Shiraz University of Medical Sciences). Serum samples were collected in two Eppendorf tubes, and stored at − 70 °C. Biochemical assessments were described previously [27]. Briefly, serum albumin, BUN, creatinine, Fasting Blood Sugar (FBS), and lipid profile (Triglyceride (TG), Low-Density Lipoprotein (LDL), High-Density Lipoprotein (HDL), and total cholesterol), were assessed by calorimetric assays using Biosystem SA auto-chemistry analyzer (DIRUI CS-T240, Spain).

### DNA extraction and SNP genotyping

Blood samples for genotyping were collected after 10–12 h overnight fasting in tubes containing EDTA as an anticoagulant and kept at − 70 ∘C until extraction. Genomic DNA was extracted from the whole blood by Cinnagen Kit DNPTM protocol (DNG plus DNA Extraction Kit, Cinnagen Company, Tehran, Iran). All the polymorphisms were evaluated by PCR–RFLP technique. TP53 Arg72Pro polymorphism (rs1042522) was identified by amplifying genomic DNA with the forward primer 5'-TCTGGTAAGGACAAGGGTTGG-3' and the reverse primer 5'-GGAAGGGACAGAAGATGACAG-3'. PCR sets for amplification of rs1042522 were 94◦C for 5 min (pre-denaturation), 35 cycles of 95◦C for 30 (denaturation), 59◦C for 30 s (annealing), and 72◦C for 30 s (extension), and completed with a 5-min final extension at 72◦C. PCR products were digested with *BstU*1 and electrophoresed on 4% polyacrylamide gels, stained with ethidium bromide and determined using a video gel documentation system (Vilber Lourmat, Marne la-Valle´e, France). A single 300 bp band is obtained for Proline homozygotes (Pro/Pro), whereas the cleaved fragment, homozygous for Arginine (Arg/Arg), gave rise to 220- and 80-bp fragments. All three bands were observed for heterozygotes (Arg/Pro).

The 16-bp Ins/Del polymorphism was detected by PCR with the following primers:

Forward:5′CTGAAAACAACGTTCTGGTA3′

Reverse:5′AGGGGGACTGTAGATGGGTG3′

PCR setting for amplification of rs1042522 were 94◦C for 5 min (pre-denaturation), 35 cycles of 95◦C for 30 (denaturation), 60◦C for 30 s (annealing), and 72◦C for 30 s (extension), and completed with a 5-min final extension at 72◦C.

Wild type alleles, designated Del allele (no duplication) resulted in 119 bp fragment and the variant alleles, designated Ins allele (with 16 bp duplication) resulted in 135 bp fragment.

### Statistical analyses

In this study continuous variables were reported as a mean and standard deviation and differences between groups were examined by independent sample t-test. Besides, nonparametric data were reported by median and interquartile range (IQR) and tested by Mann–Whitney U test. The Categorical variables were reported as a number and percentage and chi-square (χ2) test used to test the difference between groups. In addition, ANOVA test was used for comparing anthropometric parameters and biochemical variable differences between genotype and haplotype groups. The odds ratio (OR) with its 95% confidence interval (CI) was calculated through logistic regression analysis (adjusted for age and sex) to measure the association between genotypes and haplotypes of the two polymorphisms and sarcopenia. The independent t-test was used to compare the anthropometric and biochemical variable differences between the sarcopenic and non-sarcopenic participants. Data were analyzed using SPSS 25.0 (SPSS Inc., Chicago, USA) and *P*-values < 0.05. were considered statistically significant.

## Results

### Basic characteristics

Comparison of age, anthropometric data, body composition data and biochemical parameters between sarcopenic patients and control were presented in Table 1. There were significant differences between sarcopenic and control in the cases of age (*P* = 0.004), intracellular water (*P* < 0.001), extracellular water (*P* < 0.001), total body water (TBW) (*P* < 0.001), Body Mass Index (BMI) (*P* < 0.001), total skeletal muscle mass (*P* < 0.001), body fat (*P* < 0.001), skeletal muscle mass index (SMI) (*P* < 0.001), Appendicular lean mass (ALM) (*P* < 0.001), hand grip strength (*P* = 0.001), gait speed (GS) (*P* < 0.001), GLU (*P* = 0.01), albumin (*P* = 0.001), creatinine (*P* = 0.013), calcium (*P* = 0.026) and triglyceride level (*P* = 0.038).

**Table 1:** Comparison of the sarcopenic, sarcopenic obesity and healthy participants regarding baseline characteristics

Variable	Controls (*n* = 189)	Sarcopenia (*n* = 65)	*P* value
Age	69.00 (5)	71.00 (8)	**0.004**
Intracellular water	20.400 (5.8)	18.750 (4.4)	** < 0.001**
Extracellular water	12.800 (3.6)	11.750 (2.8)	** < 0.001**
Total body water	33.250 (9.5)	30.350 (7.1)	** < 0.001**
BMI, Kg/m^2^	28.15 ± 4.48	23.46 ± 3.16	** < 0.001**
Total Skeletal Muscle Mass, kg	25.263 (7.6)	22.400 (5.7)	** < 0.001**
Body fat, kg	26.23 ± 8.38	19.16 ± 6.73	** < 0.001**
Skeletal muscle mass index (SMI), Kg/m^2^	7.45 ± 0.81	6.28 ± 0.72	** < 0.001**
Appendicular lean mass (ALM), kg	18.7300 (6.57)	17.1500 (5.35)	** < 0.001**
Handgrip Strength, kg	41.6667 (31.04)	33.7500 (17.92)	**0.001**
Gait speed m/s	0.8300 (0.23)	0.7200 (0.12)	** < 0.001**
GLU	104.00 (18)	99.50 (18)	**0.010**
Albumin, g/dl	4.000 (0.3)	3.800 (0.4)	**0.001**
Creatinine, mg/dl	0.850 (0.1)	0.900 (0.2)	**0.013**
Calcium	9.300 (0.6)	9.100 (0.7)	**0.026**
BUN, mg/dl	14.00 (4)	14.00 (4)	0.193
FBS, mg/dl	94.0000 (18.00)	89.5000 (18.00)	0.010
Cholesterol, mg/dl	180.00 (63)	183.50 (46)	0.743
LDL-C, g/dl	97.50 (49)	105.50 (58)	0.241
HDL-C, mg/dl	49.00 (13)	50.50 (18)	0.546
Triglyceride, mg/dl	131.50 (76)	112.00 (63)	**0.038**

### Genotype and allele frequency of TP53

Table 2 presents genotype and allele frequencies of TP53 polymorphisms of the codon 72 polymorphism in exon 4 (rs1042522) and Intron 3 16 bp Del/Ins (rs17878362) polymorphism (rs17878362) and haplotypes and their association with sarcopenia. For the codon 72 polymorphism, the heterozygous Arg/Pro was the most frequent genotype in sarcopenic group and Pro/Pro was the most prevalent genotype in the control group. In the case of codon 72 polymorphism in exon 4, the genotype frequency of Arg/Arg, Arg/Pro and Pro/Pro in sarcopenia patients were 23.4%, 26.5% and 48.1% respectively. Besides, the genotype frequency of Arg/Arg, Arg/Pro and Pro/Pro in controls were 40.0%, 47.7%, 12.3% respectively. Minor allele frequency in sarcopenic group was 39%. In the co-dominant model, it was observed that Arg/Pro genotype carriers were at a lower (OR_adj_ = 0.175, 95% CI = 0.068 – 0.447; *P* < 0.001) risk of sarcopenia compared to the respective control group. In the dominant model, the proline carriers had also lower risk for sarcopenia (OR_Adj_: 0.515, 95% CI = 0.269–0.986; *P* = 0.045). In the recessive model, Pro/Pro genotype carriers were at (OR_Adj=_ 0.147, 95% CI = 0.064 – 0.337; *P* < 0.001) decreased risk of sarcopenia compared to the respective control group.

**Table 2:** Association of genotypes of TP53 codon 72 polymorphism in exon 4 and Intron 3 16 bp duplication polymorphism and haplotypes with sarcopenia

Polymorphism	Genotype / Haplotype	Controls (*n* = 189)	Sarcopenia patients (*n* = 65)	*P* value	OR (95% CI)	*P*_Adj_ value (age, sex)	OR_Adj_ (95% CI)
**Codon 72**	Co-Dominant model						
	Arg/Arg	48 (25.4)	26 (40%)	-	Reference	-	Reference
	Arg/Pro	50 (26.5)	31(47.7%)	** < 0.001**	0.18 (0.072 – 0.449)	** < 0.001**	0.17 (0.068 – 0.447)
	Pro/Pro	91 (48.1)	8 (12.3%)	0.750	1.11 (0.565 – 2.211)	0.893	1.050 (0.518 – 2.126)
	Dominant model						
	Arg/Arg	48 (25.4)	26 (40%)	-	Reference	-	Reference
	Arg/Pro + Pro/Pro	141 (74.6)	39 (60%)	**0.047**	0.53 (0.284–0.992)	**0.045**	0.51 (0.269 -0.986)
	Recessive model						
	Arg/Arg + Arg/Pro	98 (51.9)	57 (87.7%)	-	Reference	-	Reference
	Pro/Pro	91 (48.1)	8 (12.3%)	** < 0.001**	0.15 (0.068 – 0.334)	** < 0.001**	0.14 (0.064—0.337)
**Allele**	Arg	146 (38.6)	83 (68%)				
	Pro	232 (61.4)	39 (32%)				
**16-bp duplication**	Dominant model						
	Del/Del	122 (64.6)	50 (76.9%)	-	Reference	-	Reference
	Del/Ins + Ins/Ins	67 (35.4)	15 (23.1%)	0.223	1.52 (0.775 – 2.982)	0.311	1.42 (0.716 – 2.852)
**Allele**	Del						
	Ins						
Haplotypes	Arg-Del	43 (22.8)	25 (38.5%)	-	Reference	-	Reference
	Pro-Del	79 (41.8)	25 (38.5%)	0.342	0.34 (0.038 – 3.114)	0.210	0.36 (0.038 – 3.438)
	Arg-Ins	5 (2.6)	1 (1.5%)	0.074	0.54 (0.279 – 1.061)	**0.043**	0.48 (0.231 – 1.011)
	Pro-Ins	62 (32.8)	14 (21.5%)	**0.015**	0.38 (0.181 – 0.831)	**0.022**	0.47 (0.210 – 1.068)

Regarding the 16-bp duplication polymorphism, the homozygous Del/Del was the most frequent genotype in both groups. The Del/Del genotype (no duplication) frequency in sarcopenia patients was 64.6% and Del/Ins + Ins/Ins genotype (with at least one duplication) frequency was 35.4%. In controls, the genotype frequency of Del/Del was 76.9% and genotype frequency of Del/Ins + Ins/Ins was 23.1%. No statistically significant difference was observed when analyzed using dominant model. Also, there was no significant association with Intron 3 16-bp Del/Ins (rs17878362) polymorphisms and the risk of sarcopenia.

The most frequent haplotype in sarcopenia and control groups was Pro-Del. Finally, comparing the common TP53 Arg-Del haplotype (reference) with the other expected haplotypes showed that the carriers of Arg-Ins (OR_adj_: 0.484, 95% CI = 0.231 – 1.011, *P* = 0.043) and Pro-Ins (OR_adj_: 0.473, 95% CI = 0.210 – 1.068, *P* = 0.022) haplotypes decreased the risk of developing sarcopenia**.**

### The Effect of Tp53 polymorphisms and haplotypes on body composition and anthropometric parameters

The associations of the TP53 polymorphisms and their haplotypes with body composition, anthropometric in the studied population were analyzed (Table 3). In the case of codon 72 polymorphisms in exon 4, total skeletal muscle mass (*P* = 0.03), ALM (*P* = 0.04), SMI (*P* = 0.007) and hand grip strength (*P* = 0.01) were significantly different between genotypes. Arg/Arg genotype displayed the lowest value of total skeletal muscle mass, ALM, SMI among all genotypes. For intron 3 16-bp Del/Ins (rs17878362), no association between body composition and anthropometric parameters were observed between genotype groups. Total skeletal muscle mass (*P* = 0.01), SMI (*P* = 0.02) and ALM (*P* = 0.01) were significantly different between haplotypes. Arg/Del haplotype had the lowest values and Arg/Ins had the highest values.

**Table 3:** Association of genotypes of TP53 codon 72 polymorphism in exon 4 and Intron 3 16 bp duplication polymorphism and their haplotypes with anthropometric, body composition and biochemical parameters

Variable	Exon 4 codon 72			*P* value	16-bp insertion/deletion		*P* value	Haplotypes				*P* value
	Arg/Arg	Arg/Pro	Pro/Pro		Ins/Ins + Ins/Del	Del/Del		Arg-del	Arg-Ins	Pro-del	Pro-Ins	
**Total Skeletal Muscle Mass, kg**	23.50 ± 5	24.170 ± 4.4	25.34 ± 4.7	**0.03**	24.12 ± 4.23	24.57 ± 5	0.48	23.22 ± 4.7	26.66 ± 7.3	25.46 ± 5	23.92 ± 3.8	**0.01**
**Appendicular Lean Mass, Kg**	17.72 ± 4.2	18.36 ± 3.91	19.29 ± 4.02	**0.04**	18.25 ± 3.53	18.67 ± 4.3	0.44	17.51 ± 4.04	20.10 ± 5.86	19.43 ± 4.3	18.11 ± 3.30	**0.01**
**Total Body Fat Mass, Kg**	23.38 ± 8.2	24.92 ± 9.76	24.79 ± 7.7	0.46	24.48 ± 8.67	24.39 ± 8.5	0.93	23.23 ± 23	25.10 ± 12.53	25.15 ± 8.8	24.43 ± 8.42	0.55
**Skeletal Muscle Mass Index (SMI), Kg/m**^**2**^	6.95 ± 0.95	7.06 ± 0.98	7.37 ± 0.84	**0.007**	7.16 ± 0.85	7.14 ± 0.97	0.91	6.89 ± 0.91	7.57 ± 1.22	7.31 ± 0.98	7.12 ± 0.81	**0.02**
**Handgrip strength (MS), Kg**	40.86 ± 19	39.26 ± 15.95	46.74 ± 18.4	**0.01**	44.84 ± 17.7	41.58 ± 18.2	0.18	40.09 ± 18.95	49.44 ± 19.01	42.56 ± 17.7	44.48 ± 17.72	0.39
**Gait Speed (GS), m/s**	0.78 ± 0.29	0.78 ± 0.20	0.84 ± 0.19	0.05	0.84 ± 0.20	0.79 ± 0.20	0.64	0.85 ± .0.18	0.80 ± 0.20	0.77 ± 0.24	0.78 ± 0.19	0.22
**GLU**	109.67 ± 30.54	114.40 ± 40.7	110.83 ± 35.19	0.69	111.13 ± 39.6	111.87 ± 33.76	0.87	111.92 ± 41.06	112.87 ± 25.21	101.33 ± 5.7	110.42 ± 31.75	0.875
**Albumin, g/dl**	3.94 ± 0.24	3.92 ± 0.23	3.94 ± 0.22	0.77	3.99 ± 0.22	3.91 ± 0.24	**0.02**	3.93 ± 025	4.10 ± 0.141	3.90 ± 0.23	3.98 ± 0.23	0.06
**Creatinine, mg/dl**	0.87 ± 0.13	0.94 ± 0.35	0.89 ± 0.15	0.13	0.93 ± 0.36	0.89 ± 0.16	0.29	0.87 ± 0.14	0.87 ± 0.08	0.91 ± 0.17	0.93 ± 0.35	0.47
**Calcium**	9.31 ± 0.55	9.32 ± 0.66	9.27 ± 0.42	0.78	9.41 ± 0.55	9.25 ± 0.54	0.03	9.28 ± 0.54	9.63 ± 0.69	9.23 ± 0.54	9.39 ± 0.54	0.10
**BUN, mg/dl**	14.41 ± 4.01	15.95 ± 7.4	14.45 ± 4.89	0.14	15.11 ± 7.29	14.82 ± 4.64	0.70	14.43 ± 4.15	14.17 ± 2.23	15.09 ± 4.9	15.19 ± 7.5	08.35
**FBS, mg/dl**	99.67 ± 30.54	104.39 ± 40.7	100.82 ± 35.19	0.694	101.86 ± 33.7	101.12 ± 39.60	0.879	100.41 ± 31.75	91.33 ± 5.75	102.86 ± 35.2	101.95 ± 41.06	0.875
**Cholesterol, mg/dl**	182.04 ± 43.84	178.64 ± 42.6	190.95 ± 43.64	0.157	194.20 ± 44.6	179.54 ± 42.12	**0.013**	180.88 ± 43.43	195.00 ± 50.54	178.62 ± 41.3	194.14 ± 44.52	0.100
**LDL-C, g/dl**	100.27 ± 34.80	98.31 ± 33.8	109.37 ± 34.03	**0.080**	109.45 ± 16.1	100.02 ± 33.49	**0.045**	99.37 ± 34.25	110.33 ± 42.8	100.46 ± 33.1	109.38 ± 25.85	0.258
**HDL-C, mg/dl**	53.58 ± 12.95	49.72 ± 12.5	49.74 ± 12.89	0.102	51.94 ± 13.05	50.37 ± 12.79	0.372	53.07 ± 13.15	59.17 ± 9.62	48.49 ± 12.26	51.35 ± 13.16	**0.048**
**Triglyceride, mg/dl**	126.78 ± 62.16	154.19 ± 85.1	157.55 ± 74.19	**0.020**	159.04 ± 86.1	141.53 ± 68.42	0.089	125.84 ± 61.91	137.33 ± 70.07	152.37 ± 72.5	160.80 ± 87.44	**0.039**

### The Effect of Tp53 polymorphisms and their haplotypes on biochemical parameters

The effect of Tp53 polymorphisms and haplotypes on biochemical parameters was presented in Table 3. TG was significantly different between genotypes of exon 4 codon 72 (*P* = 0.02). Arg-Arg genotype had the lowest value of TG (126.78 ± 62.16) among exon 4 codon 72 genotypes. Albumin (*P* = 0.02), cholesterol (*P* = 0.013), and LDL (*P* = 0.045) were different between intron 3 16-bp Del/Ins (rs17878362) genotype groups. In addition, for haplotypes analysis, HDL (*P* = 0.048) and TG (*P* = 0.039) were significantly different between haplotype groups.

## Discussion

The objective of our study is to investigate the association TP53 exon 4 Arg72pro (rs1042522) and Intron 3 16-bp Del/Ins (rs17878362) polymorphisms and their haplotypes with sarcopenia, anthropometric, body composition and biochemical parameters in Iranian older adults. The most remarkable finding of this study is that codon 72 (Pro72Arg) polymorphism in exon 4 is associated with susceptibility to sarcopenia. Moreover, in haplotype analysis, haplotypes of Arg-Ins and Pro-Ins were associated with decreased risk of sarcopenia compared to Arg-Del haplotype. We did not observe statistically significant differences between the Intron 3 16-bp Del/Ins polymorphism (rs17878362) and sarcopenia risk.

There is controversy whether the p53 is involved in skeletal muscle atrophy or it is necessary for normal muscle function [29]. Aging is associated with increased expression of p53 and canonical p53 target genes in skeletal muscle. Furthermore, in young adult mice p53 expression in skeletal muscle fibers induces skeletal muscle atrophy and is necessary for at least one acute form of skeletal muscle atrophy [30]. Recent literature focuses attention on responsibility of p53 in a wide array of signaling pathways in skeletal muscle including activating pathways that increase time for cell repair, cell cycle arrest and autophagy, initiating DNA fragmentation, and inducing apoptosis and accordingly preventing cell proliferation and mitochondrial function [31–33]. Regarding to the underlying pathophysiological mechanisms of sarcopenia, mitochondrial dysfunction, altered apoptotic and autophagic signaling were frequently reported [34]. Our observations confirmed with previous results. Shafiee et al. 2018 introduced p53 as one of the most significant hub genes, which may be involved in aging muscle and sarcopenia [14]. Besides, the observed association of the codon 72 polymorphism in exon 4 with sarcopenia supported by the findings of Di Renzo et al. [20]. They reported that Arg/Arg genotype increases the sarcopenia risk up to 20%, which is in line with the findings of our study.

The haplotype effect of these two common polymorphisms of TP53 has been widely studied and the linkage disequilibrium between TP53 polymorphisms region could be an important risk factor affecting the incidence of sarcopenia [35]. Previous works have only focused on the association of haplotype effect of the two polymorphisms on various cancer susceptibility [35, 36]. In the present study, we observed that Pro-Ins haplotype decreased the risk of sarcopenia compared to Arg-Del haplotype.

Another intriguing result was the effect of TP53 polymorphisms and their haplotypes on body composition and anthropometric parameters. In the case of codon 72 polymorphisms in exon 4, total skeletal muscle mass, ALM, SMI and hand grip strength were significantly different between genotypes. For the haplotypes, total skeletal muscle mass, SMI and ALM were significantly different between haplotypes. According to our best knowledge, there is no study on the association between TP53 polymorphisms and skeletal muscle traits, however, a polymorphism study of TP53 gene on Saudi population observed that codon 72 polymorphism of TP53 increased BMI and waist/hip ratio [37]. Saleem et al. [38] provided the first report to demonstrate that p53 modulates skeletal muscle mitochondrial content and function. In contrast, Ebert et al. proposed that p53 activity within skeletal muscle fibers is not essential for age-related skeletal muscle atrophy or weakness [29]. Mouse models with constitutively active p53 alleles imply that the increase in p53 activity is associated with cell and tissue aging [39]. In current work we found that individuals with Arg/Arg genotype displayed the lowest value of total skeletal muscle mass, ALM, SMI and hand grip strength. Since protein with Arg72 was reported to have up to 15-fold increased apoptotic activity compared to Pro72 variant, the possible explanation for lower values of total skeletal muscle mass, ALM, SMI and hand grip strength in Arg/Arg genotype is that, similar to previous mouse models increased p53 activity in Arg carriers resulted in loss of muscle mass.

Furthermore, studying the effect of Tp53 polymorphisms and their haplotypes on biochemical parameters revealed that codon 72 polymorphism in exon 4 significantly affect TG values. Compelling evidence suggests the fundamental role of p53 in adipose tissue metabolism and homeostasis, insulin resistance and development of metabolic diseases such as type 2 diabetes [40], cardiovascular diseases [41] and obesity [42]. A study on Brazilian population reported that Arg allele carriers showed lower HDL levels and a higher frequency of cardiovascular disease than Pro allele subjects [41]. Similarly, in our study, we found that carriers of the Arg allele had lower levels of TG. One possible explanation is that the higher TP53 apoptotic activity of Arg carriers negatively impact adipogenesis which results in lower TG value. In spite of a significantly higher frequency of the p53 Arg72 allele in patients with premature coronary artery disease, the levels of total cholesterol, LDL, HDL, TG, were not significantly influenced by the p53 genotypic variants [43]. Sabir et al. [37] reported that the Arg allele of p53 variant has shown significant influence on cholesterol, LDL level, and random insulin levels in obese subjects. Regarding metabolic function of this gene, our results also share similarities with Murphy et al. used a humanized knock-in mouse model [44] and found that codon 72 polymorphism of TP53 has a significant impact on the metabolic response to a high-fat diet. In addition, Shafiee et al. [14] reported that P53 is involved in lipid storage in older women.

For Intron 3 16-bp Del/Ins polymorphism, albumin, calcium, cholesterol, and LDL were different between genotype groups. Since it was reported that elevated expression and activity of p53 is well known to occur in the adipocytes, decreased expression and activity of P53 as a result of functional mutation may lead to increased rates of adipogenesis [45], which confirmed in our study. Gemignani et al. reported that the 16-bp insert allele was associated with lower levels of TP53 transcripts [25]. We observed that individuals with 16-bp insertion showed a higher value of LDL and cholesterol. It seems that decreasing the expression of TP53 in individuals with 16-bp insertion results in increasing the adipogenesis.

In regard to the haplotypes analysis, HDL and TG were significantly varying between haplotypes. Arg-Ins genotypes showed higher values of HDL. Pro-Ins genotypes had higher values of TG. The results of SNP combination proved the findings for each SNP alone. Individuals with Pro allele and 16-bp insertion revealed higher lipid values when analyzed independently. After combination in haplotype form, we found that carriers of Pro-Ins had greater lipid values.

The limitation of this study is the relatively small sample size that may affect the statistical power of associations of the codon 72 polymorphism in exon 4 and intron 3 16 bp Del/Ins with the risk of sarcopenia. Participants registered in the current study were nominated from a cohort study from the South of Iran and may not be reflected as the general population of Iranian old adults. As a strength, this is a first study of association of the codon 72 polymorphism in exon 4 and intron 3 16-bp Del/Ins with risk of sarcopenia. Since, Race and ethnicity may clarify some of the high variability of occurrence frequency for sarcopenia, and it is established that body composition varies between major races, finding the genetic factor that influence the susceptibility to these two traits in diverse populations is essential. So that, as another strength, in this study for the first time the association of these polymorphisms with the risk of sarcopenia in Iranian population was inspected.

## Conclusions

This study has gone some way towards enhancing our understanding of the role of the *TP53* gene in muscle traits, muscle function and susceptibility for sarcopenia. For the first time we reported that codon 72 (Pro72Arg) polymorphism in exon 4, and Arg/Ins and Pro/Ins haplotypes could decrease the susceptibility to sarcopenia in Iranian older adults. No significant relation between intron 3 TP53 polymorphisms and susceptibility to sarcopenia was detected. In addition, the effect of TP53 genetic variants and their haplotypes on LDL and TG was observed. We hope that our research will serve as a base for future studies on this subject.

## Data Availability

The datasets generated during and analyzed during the current study are not publicly available due to [according to protect the patient information] but are available from the corresponding author on reasonable request.

## Abbreviations

PCR: RFLP
ALM: Appendicular Lean Mass
SMI: Skeletal Muscle Mass Index
ACE: Angiotensin Converting Enzyme
ACTN3: α-Actinin-3
AR: Androgen receptor
BMI: Body Mass Index
CI: Confidence interval
CNTF: Ciliary Neurotrophic Factor
FBS: Fasting Blood Sugar
GS: Gait Speed
HDL: High-Density Lipoprotein
HGS: Hand grip Strength
IGF-1: Insulin Growth Factor 1
IL-6: Interleukin-6
IQR: Interquartile Range
LDL: Low-Density Lipoprotein
MSTN: Myostatin-Related Genes
OR: Odds Ratio
TG: Triglyceride
TP53: Tumor Suppressor Protein 53
TRHR: Thyrotropin-Releasing Hormone Receptor
VDR: Vitamin D receptor
